# Polyp segmentation based on implicit edge-guided cross-layer fusion networks

**DOI:** 10.1038/s41598-024-62331-5

**Published:** 2024-05-22

**Authors:** Junqing Liu, Weiwei Zhang, Yong Liu, Qinghe Zhang

**Affiliations:** 1 Hubei Engineering and Technology Research Center for Construction Quality Inspection Equipment, China Three Gorges University, Yichang, 443002 Hubei People’s Republic of China; 2 College of Computer and Information Technology, China Three Gorges University, Yichang, 443002 Hubei People’s Republic of China

**Keywords:** Polyp segmentation, Implicit edge, Feature fusion, Multi-scale feature reasoning, Colonoscopy, Image processing

## Abstract

Polyps are abnormal tissue clumps growing primarily on the inner linings of the gastrointestinal tract. While such clumps are generally harmless, they can potentially evolve into pathological tumors, and thus require long-term observation and monitoring. Polyp segmentation in gastrointestinal endoscopy images is an important stage for polyp monitoring and subsequent treatment. However, this segmentation task faces multiple challenges: the low contrast of the polyp boundaries, the varied polyp appearance, and the co-occurrence of multiple polyps. So, in this paper, an implicit edge-guided cross-layer fusion network (IECFNet) is proposed for polyp segmentation. The codec pair is used to generate an initial saliency map, the implicit edge-enhanced context attention module aggregates the feature graph output from the encoding and decoding to generate the rough prediction, and the multi-scale feature reasoning module is used to generate final predictions. Polyp segmentation experiments have been conducted on five popular polyp image datasets (Kvasir, CVC-ClinicDB, ETIS, CVC-ColonDB, and CVC-300), and the experimental results show that the proposed method significantly outperforms a conventional method, especially with an accuracy margin of 7.9% on the ETIS dataset.

## Introduction

Medical image segmentation is one of the key stages in medical image analysis, where regions of interest (such as tumors, organs, blood vessels, and other structures) are identified in medical image data^1^. Segmentation is particularly important in locating polyps, which are abnormal tissue clumps that grow on mucosal surfaces of the human body. While polyps are mostly benign, some of them might be cancerous, and so long-term regular polyp monitoring is necessary. This monitoring process crucially depends on the accuracy of polyp segmentation for early diagnosis of polyp diseases^2^.

The main components of colon polyps are the intestinal mucosa, submucosa and muscularis propria, which are usually bounded by the surrounding normal mucosa. Depending on the tissue structure and morphology, colon polyps can be divided into different types, of which the most common is adenomatous polyp. Adenomatous polyps are formed by the proliferation of glandular epithelial cells and may sometimes develop into malignant lesions, which are one of the main precursor lesions of colorectal cancer. Colonoscopy allows the physician to directly visualize mucosa, blood vessels, lesions and foreign bodies in the colon, and to perform biopsies or resections to obtain tissue samples for pathology. Accurate polyp segmentation is a challenging task for two main reasons: (I) polyps show wide variability in size and color; (II) the boundaries between polyps and their surrounding mucosa are quite blurry and of low contrast^2^.

Most existing attention-based segmentation methods are designed to enhance the model's attention capability and flexibility^3^. However, the adoption of attention mechanism methods can lead to problems such as high computational complexity, poor generalization ability, overfitting risks, and sensitivity to data skewness. Zhou et al.^4^ utilized a context auto-regressive attention mechanism to address these issues. By combining the context auto-regression method, the model could better consider previously generated structural information during neural architecture search. They integrated full attention and context auto-regression to construct a full attention-based neural architecture search framework, significantly increasing the computational complexity of the model. However, the introduction of full attention mechanism also leads to the problem of the model's over-reliance on local information, thereby increasing the risk of overfitting, which becomes more prominent when training data is insufficient or noisy. Tan et al.^5^ proposed the EfficientDet V2 model, which adopted a self-attention mechanism to enhance the model's attention capability on input features and reduce redundant computations. EfficientDet V2 introduced self-attention mechanism to dynamically adjust the feature correlations between different positions. The introduction of self-attention modules increases the training difficulty of the model, as additional parameter tuning and hyperparameter adjustments are required to ensure model stability and convergence. This paper addresses these issues by improving the context attention mechanism through edge guidance, solving the problem of the model's over-reliance on local information. It also utilizes a multi-scale inference module to detect fused multi-scale image features, thereby improving the model's generalization and stability.

In recent years, deep-learning-based methods have led to significant progress in polyp segmentation^4^. These methods use deep neural networks to learn more discriminative feature representations from endoscopic polyp images. However, since bounding-box detectors are usually used for polyp detection, polyp boundaries can’t be accurately located. To address this issue, Brandao et al.^6^ used fully convolutional networks (FCN) with pre-trained models to identify and segment polyps. Qadir et al.^7^ proposed a method utilizing Fully Convolutional Neural Networks (FCNNs) to predict 2D Gaussian shapes, aiming to achieve faster detection speeds by employing the FCNN model for polyp detection. Subsequently, good polyp segmentation performance was achieved by a U-Net^8^ architecture, which mainly consisted of a contracting path to capture context and a symmetric expanding path for precise localization. However, these methods focus on segmenting the entire polyp regions, but ignore the region boundary constraints. So, region and boundary constraints were jointly utilized in Psi-Net^9^ for polyp segmentation, but still the region-boundary relations were not fully exploited. The PolypSegNet^10^ proposed by Mahmud et al. focuses on introducing an improved encoder-decoder architecture for automating the segmentation of polyps from colonoscopy images. Guo et al.^11^ proposed a confidence-aware resampling method aimed at addressing non-equivalent images and pixels issues in polyp segmentation tasks. Through meta-learning mixup techniques, the method aims to enhance the model's generalization across different samples.In addition, Fan et al*.*^2^ proposed the parallel reverse attention network (PraNet) model based on the deep salient object detection network proposed by Chen et al*.*^12^. While the PraNet model has generally demonstrated remarkable segmentation performance, its effectiveness in solving multi-scale problems is still limited.

To address the limitations of the aforementioned polyp segmentation methods, a new implicit-edge-guided cross-layer fusion network is introduced in this paper. This network focuses on uncertain regions of saliency that are highly correlated with polyp boundaries, and these saliency regions are used as attention maps in the proposed network to extract refined low-level features. Finally, multi-scale feature reasoning is employed to detect and fuse different multi-scale image features, and thereby obtain final polyp segmentation outcomes. The key contributions of this paper are as follows:

i. A new deep network model is proposed for polyp segmentation. This model enhances the segmentation outcomes by effectively exploiting global contextual information, cross-level feature fusion, low-level feature refinement, and multi-scale feature inference.
ii. In order to expand the spatial receptive field of the backbone network, an attention encoding–decoding pair is proposed for the receptive-field coordinates.
iii. To compensate for the absence of explicit shape boundary information, an implicit-edge-enhanced contextual attention module is designed based on multi-headed self-attention and edge information.
iv. A multi-scale feature reasoning module is proposed to refine the low-level features with the rough prediction maps obtained from the high-level fused features, and thereby obtain final segmentation outcomes.

The remainder of this paper is organized as follows. Firstly, related work on automated polyp segmentation methods is briefly reviewed in “Related work” section. Then, the proposed polyp segmentation model and each of its modules are explained in detail in “Method” section. Thus, “Experimental setup and results” section highlights the experimental setup and the results of the experiments, an ablation study, and comparative analysis. Finally, conclusions are made in “Conclusion” section.

## Related work

In this section, we briefly review the literature on existing related methods of semantic segmentation, salient object detection, and context-aware deep learning.

### Semantic segmentation

In a semantic segmentation task, each image pixel should be labelled with the most likely semantic class. With the recent emergence of deep learning methods, these methods have gradually become the mainstream ones for semantic segmentation. For example, U-Net is a semantic segmentation model based on convolutional neural networks. This model essentially employs a symmetric encoder-decoder structure and introduces jump connections to boost segmentation performance. In addition, a mask R-CNN^13,14^ jointly detects objects and performs semantic segmentation. A dual attention network^15^ employs a self-attention mechanism and a spatial-channel dual-branch network for local and global feature fusion. EfficientNet is an efficient neural network architecture that achieves good performance in semantic segmentation by scaling the network width, depth, and resolution, even when computational resources are limited. HRNet is a multi-scale, high-resolution neural network structure. It maintains information flow at various resolutions by parallelly connecting multiple feature maps and constructs dense feature representations at each resolution. This design enables HRNet to effectively capture semantic information at different scales, leading to significant performance improvements in tasks like image segmentation tasks.

### Salient object detection

Instead of locating and classifying entire image regions, salient object detection (SOD)^16^ focuses on identifying the most important target objects or regions. Unlike semantic segmentation, SOD does not employ simple powerful baseline models. Instead, the state-of-the-art SOD approaches use object boundary regions as supplementary information to improve the saliency estimation quality through multi-task learning strategies. One of the most prominent SOD approaches employs an edge-guided network (EGNet)^17^, where a bottom-up edge detection branch and a side-out fusion strategy are used towards top-down aggregation of salient object branches. Alternatively, a boundary-aware network (BANet)^18^ performs side-out fusion on boundary branches, while only a single stream is used for object branches. However, BANet does not treat edge detection as a separate task, but rather combines edge and target detection results for saliency map generation. All these methods led to competitive experimental results, and thus demonstrated the usefulness of edge guidance for obtaining reliable object representations. However, the complexity of edge detection is generally high, and edge detectors (such as the Canny edge detector^6^) usually produce redundant edges that are unrelated to the object of interest. For more accurate segmentation, self-attention^12^ considers predicted inverse regions and captures saliency details.

The above approaches inspired the following intuitive idea: without explicit edge guidance, edge-related contextual information can be alternately obtained from saliency maps. To realize this idea, we create uncertainty regions without explicit edge information and design a reverse significance plot with additional implicit edge regions. Our approach does not favor neither foreground nor background implicit regions, and thus leads to effective acquisition of edge-related contextual information. In the absence of explicit edge information, we thus define uncertain regions and design reverse saliency maps with implicit edge regions.

### Contextual awareness

Contextual information can lead to significantly enhanced feature representations, and hence this type of information can play a crucial role in boosting object segmentation performance. For instance, Zhao et al.^19^ proposed the PSPNet architecture, which establishes a multi-scale representation around each image pixel to get rich contextual information. Chen et al.^20^ constructed ASPP with different dilated convolutions to capture essential contextual information. In addition, rich contextual information has been obtained through self-attention mechanisms, including those used in DANet ^15^ and CCNet^21^. The former uses non-local modules to extract contextual information^22^, while the latter uses multiple cascaded cross-attention modules to obtain dense contextual information. In addition, contextual information has been also heavily exploited for target segmentation. For example, Zhang et al.^23^used multi-scale context-aware modules to extract rich contextual features. As well, Liu et al.^24^ proposed PoolNet, a deep architecture for salient object detection based on highly relevant contextual features extracted using a pyramid structure. Furthermore, Chen et al.^25^ proposed an enhanced global context-aware segmentation method in which features containing global semantic information are transformed into multi-layer features at different stages.

## Method

In this section, the proposed IECFNet framework is holistically introduced, and then the details of its three major modules are given.

### Overall architecture

**Figure 1:**
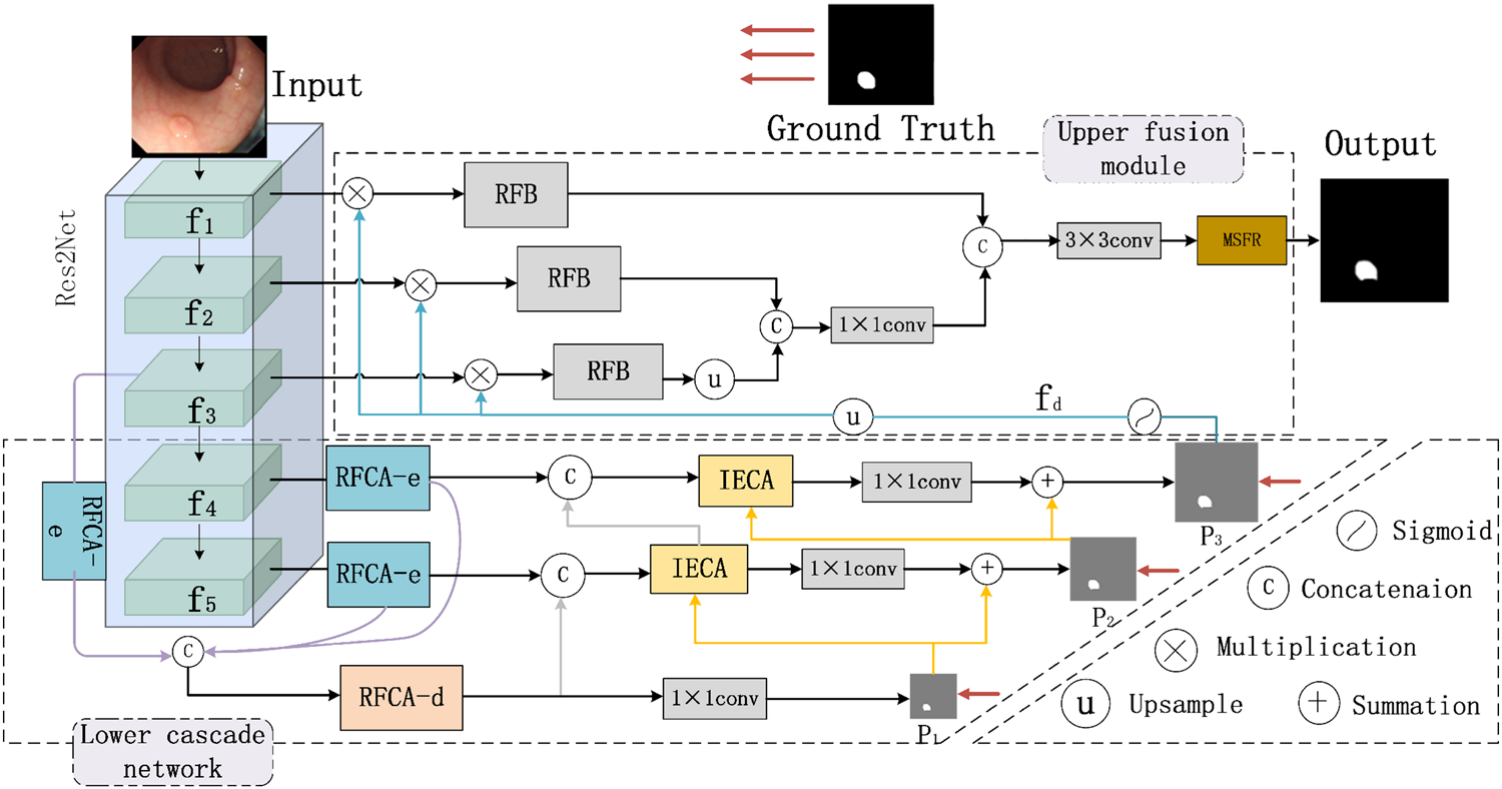
IECFNet Overall Architecture, RFCA-d and RFCA-e are used to reduce the number of channels in the input feature map, IECA implements cross-layer fusion and MSFR implements multi-scale feature inference.

As shown in Fig. 1, IECFNet consists of a backbone network as well as upper and lower hierarchical networks. In particular, a Res2Net^26^ backbone network is used to extract multi-scale features f_*i*_ (*i* = 1, 2, …, 5) from the input images. The lower cascade network gradually obtains more refined saliency maps (P_*1*_ → P_*2*_ → P_*3*_) from the bottom up. The obtained maps are thus used in the upper fusion network to get refined lower-level features. The upper fusion network first performs multi-scale feature fusion, and then carries out multi-scale feature inference to produce the final segmentation outcomes. Constructing the proposed IECFNet architecture involves the design of several modules: the receptive-field coordinate attention encoder (RFCA-e), the receptive-field coordinate attention decoder (RFCA-d), the implicit edge-enhanced context attention (IECA) module, and the multi-scale feature reasoning (MSFR) module.

As shown in Fig. 1, for the lower cascade network, the feature maps generated by the high-level backbone network are introduced into the RFCA-e modules. These modules not only expand the network receptive field, but also help reduce the computing cost by reducing the number of feature map channels. Specifically, the feature maps of the three RFCA-e modules are connected and fed to the RFCA-d module. From the bottom to the top, the RFCA-d module in the first layer of the lower cascade network predicts the initial polyp significance map (*P*_1_). In the second layer, the output feature maps from the RFCA-e and RFCA-d modules are connected and input to IECA, and *P*_1_ is used as contextual information to generate the significance map P2, which represents further contextual information. The feature map f4 generated by the fourth layer of the backbone network is input to the RFCA-e module, whose output is connected with the IECA feature map in the second layer. The convolution results are input to the IECA map in the third layer to generate the final coarse segmentation map P3. The resulting *P*_1_, *P*_2_, and *P*_3_ are compared with the Ground Truth. Binary Cross Entropy loss and Intersection over Union loss are employed, and the three calculated losses are aggregated to obtain the average value. Utilizing the average loss enables more accurate model training and accelerates convergence speed through regression calculations.

Furthermore, bilinear up-sampling is performed on *P*_3_, and the result (*f*_*d*_) is sent to the upper fusion network for low-level feature refinement. Specifically, *f*_*d*_ is multiplied with the feature maps f1, f2 and f3 respectively, and the results are sent to three receptive field blocks (RFB). Since the size of *f*_3_ is half of those of *f*_1_ and *f*_2_, it is necessary to upsample the feature map obtained after passing *f*_3_⊗*f*_*d*_ through the RFB module. Then, the output is connected with the feature map obtained after passing *f*_2_⊗*f*_*d*_ through the RFB module. Similarly, the result is connected with the feature map obtained after passing *f*_1_⊗*f*_*d*_ through the RFB module. Finally, the result is sent to the MSFR module to get the final segmentation map. The details of each of the above modules are described separately below.

### Receptive-field coordinate attention encode and decoder pair

In deep learning network models, context modules are beneficial for extracting fine-grained feature maps with high-level semantic information and low-level details. In particular, context can be essentially accounted for through self-attention mechanisms, but such mechanisms are computationally intensive. However, receptive-field coordinate attention (RFCA)^27^ can reduce the computational cost to a certain extent through performing and composing non-local operations on coordinate pairs.

**Figure 2:**
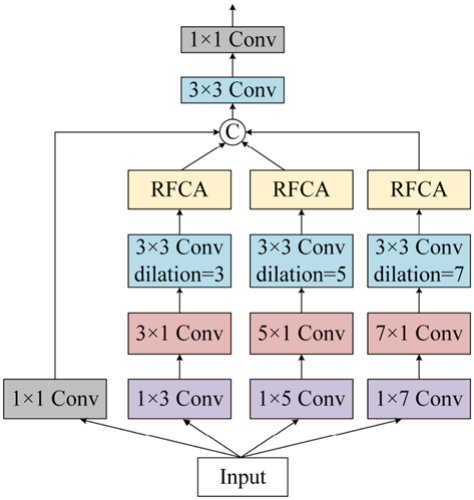
Receptive-field coordinate attention encode network structure.

Inspired by the coordinate attention mechanism, a new coordinate attention encoder is proposed (as shown in Fig. 2) based on the RFB design proposed by Song et al.^28^.

Previous studies have shown that RFCA can enhance the expressiveness of learned features in mobile networks. As shown in Fig. 2, RFCA-e aggregates low-level feature maps for bottom-up streaming, but this will inevitably increase the number of model parameters and computational complexity.

To reduce this complexity, the number of channels should be reduced without losing information details. Therefore, the RFCA-e module achieves this by employing the RFB module, expanding the receptive field via convolutions of different scales, and exploiting feature reuse and parameter sharing.

**Figure 3:**
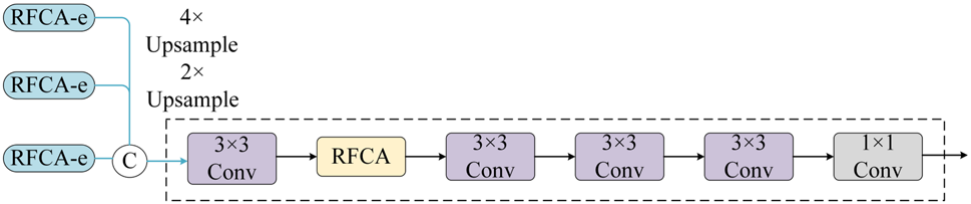
Receptive-field coordinate attention encode network structure.

Figure 3 is referred to as the receptive-field coordinate attention decode network structure, it is labeled as RFCA-d.After up-sampling the RFCA-e outputs, these outputs are concatenated along the channel dimension, and then features are extracted via convolution. The obtained features are then globally refined and relatively enriched in the RFCA module. At this point, four convolutional layers are used to obtain more enhanced features. Finally, a saliency map fused with multi-scale features is obtained.

### Implicit edge-enhanced context attention module

The performance of reverse attention^12^ in salient target detection and polyp segmentation tasks can be improved by boundary-guided SOD networks^18,29^. In such networks, using target boundaries as complementary supervision information generally improves the polyp detection accuracy. Therefore, reverse attention can be used as an effective method for implicit module.

**Figure 4:**
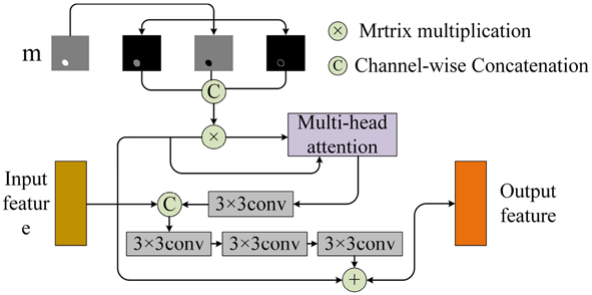
Implicit Edge-enhanced Context Attention.

Focusing on saliency and reverse saliency maps through reverse attention, boundaries generally appeared in areas with average significance scores in adjacent parts. Specifically, the average scores of boundary areas were around 0.5. Based on this observation, the saliency and reverse saliency maps could be assumed to have almost the same amount of edge information, and thus a simple subtraction operation can produce the reverse saliency map. Based on this idea, the implicit-edge-enhanced context attention (IECAM) module is proposed (as shown in Fig. 4) for extracting rich semantic features without additional boundary guidance in combination with uncertain regions.

Specifically, denote the previously obtained input saliency map by m. Also, denote the corresponding foreground map, background map, and uncertain boundary region map as mf, mb, and mu, respectively. The relationships between these maps are expressed in Eqs. (1) and (2) as follows:

1 $${m}_{f}=\text{max}\left(m-\text{0.5,0}\right),$$

2 $${m}_{b}=\text{max}\left(0.5-m,0\right),$$

3 $${m}_{u}=0.5-abs\left(m-0.5\right).$$

In Eqs. (1) and (2), the foreground and background maps are calculated using maximum values, so the corresponding regions are not only separated from each other, but also from uncertain regions. However, if Eq. (3) is used to find the uncertain boundary region map mu, redundant information can’t be easily obtained, and the computed map would be of reduced reliability.

Therefore, each pixel value is multiplied and summed with each corresponding pixel value in the input feature map X, and vector representations of the foreground, background, and uncertain region maps are calculated as follows,

4 $${w}_{f}=\sum_{i\in I}{m}_{{f}_{i}}{x}_{i},$$

5 $${w}_{b}=\sum_{i\in I}{m}_{{b}_{i}}{x}_{i},$$

6 $${w}_{u}=\sum_{i\in I}{m}_{{u}_{i}}{x}_{i},$$

where *i* ∈ *I* denotes the image pixel. As shown in Fig. 4, each vector represents the most typical feature vector in the feature space, so that *w*_*f*_ and *w*_*u*_ can effectively express the foreground and uncertain boundary regions. The pairwise inter-pixel similarity scores of the *w*_*f*_, *w*_*b*_ and *w*_*u*_ vectors (after applying the feature map xi) are calculated as follows,

7 $$\left\{\begin{array}{c}{t}_{{f}_{i}}^{{{\prime}}}={\Psi \left({x}_{i}\right)}^{T}\varnothing \left({w}_{f}\right),\\ {t}_{{b}_{i}}^{{{\prime}}}={\Psi \left({x}_{i}\right)}^{T}\varnothing \left({w}_{b}\right),\\ {t}_{{u}_{i}}^{{{\prime}}}={\Psi \left({x}_{i}\right)}^{T}\varnothing \left({w}_{u}\right).\end{array}\right.$$

8 $${s}_{{f}_{i}}=\frac{{e}^{{t}_{{f}_{i}}^{{{\prime}}}}}{N}, {s}_{{b}_{i}}=\frac{{e}^{{t}_{{b}_{i}}^{{{\prime}}}}}{N},{s}_{{u}_{i}}=\frac{{e}^{{t}_{{u}_{i}}^{{{\prime}}}}}{N},$$

9 $$\text{N}={e}^{{t}_{{f}_{i}}^{{{\prime}}}}+{e}^{{t}_{{b}_{i}}^{{{\prime}}}}+{e}^{{t}_{{u}_{i}}^{{{\prime}}}}.$$

The similarity scores ${s}_{{f}_{i}}$, ${s}_{{b}_{i}}$ and ${s}_{{u}_{i}}$ are then used to compute contextual feature maps for *w*_*f*_, *w*_*b*_ and *w*_*u*_ as follows,

10 $${t}_{i}=\delta \left({s}_{{f}_{i}}\tau \left({w}_{f}\right)+{s}_{{b}_{i}}\tau \left({w}_{b}\right)+{s}_{{u}_{i}}\tau \left({w}_{u}\right)\right).$$

where *Ψ(·)*, *Φ(·)*, *τ(·)* and *δ(·)* are pointwise convolution functions, and the value of each pixel *t*_*i*_ in the contextual map is the weighted average of the three vectors *w*_*f*_, *w*_*b*_ and *w*_*u*_.

Given an input feature *x* and a contextual feature map t, the query (*Q*), the key (*K*) and the value (*V*) are first computed using three convolutional layers:

11 $$Q={L}_{q}\cdot x, \;\; K={L}_{k}\cdot t, \;\; V={L}_{v}\cdot t,$$

where *L*_*q*_, *L*_*k*_ and *L*_*v*_ are the corresponding convolutional layer weights.

In order to achieve better generalizability, lower computational complexity, and more effective modeling of complex spatial relationships, a multi-head self-attention mechanism is used where attention is computed based on input features. With this mechanism, the feature space is divided into multiple subspaces, such that the proposed model can focus on different information aspects. Attention computation is as follows,

12 $$\left\{\begin{array}{c}{Q}_{i}=Q{W}_{i}^{Q},\\ {K}_{i}=K{W}_{i}^{K},\\ {V}_{i}=V{W}_{i}^{V},\end{array}\right.$$

13 $${head}_{i}=Attention\left({Q}_{i},{K}_{i},{V}_{i}\right), \quad i=1,\dots ,8$$

14 $$MultiHead\left(Q,K,V\right)=MH$$

15 $$MH=Concact({head}_{1},\dots ,{head}_{8}){W}^{O}$$

From Eq. (12), 8 heads are used in association with the weights W_*i*_ to form the triples *Q*_*i*_, *K*_*i*_, *V*_*i*_ (*i* = 1, …, 8). Then, the Attention weight matrix is calculated as

16 $${z}_{i}=softmax\left(\frac{{Q}_{i}{K}_{i}^{T}}{\sqrt{{d}_{k}}}\right){V}_{i}$$

Thus, each *z*_*i*_ is merged to form *Z*_*i*_, and the outcomes of the 8 headers are subsequently merged as *Z*^*C*^,

17 $${Z}^{C}=concact({Z}_{1},\dots {Z}_{8})$$

Finally, pointwise multiplication is performed via 3 × 3 convolution with *x*, and the output obtained after a series of convolution operations is summed with the corresponding pixel values of the original output *X* to obtain the output features (as shown in Fig. 4).

### Multi-scale feature reasoning

To effectively utilize multi-scale features, the output *f*_*d*_ of the IECA module is used for low-level feature refinement as shown in Fig. 1. The RFB module can expand the perceptual field, extract rich features, and reduce the computational cost. As shown in Fig. 1, *f*_*d*_ is convolved with *f*_1_, *f*_2_, and *f*_3_ to refine the three low-level feature maps, respectively. The refined feature maps are independently fed to the RFB module to get features with larger receptive fields: R(*f*_1_⊗*f*_*d*_), R(*f*_2_⊗*f*_*d*_), and R(*f*_3_⊗*f*_*d*_). Then, R(*f*_1_⊗*f*_*d*_) and R(*f*_2_⊗*f*_*d*_) are cascaded and fed to the convolution block, and the block outputs are further cascaded with R(*f*_3_⊗*f*_*d*_) and fed to a 16-channel convolution block. Finally, a multi-scale feature reasoning module utilizes low-level features and multi-scale strategies to generate the final segmentation outcomes.

**Figure 5:**
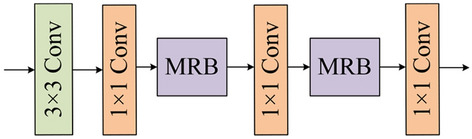
Multi-scale Feature Reasoning module.

**Figure 6:**
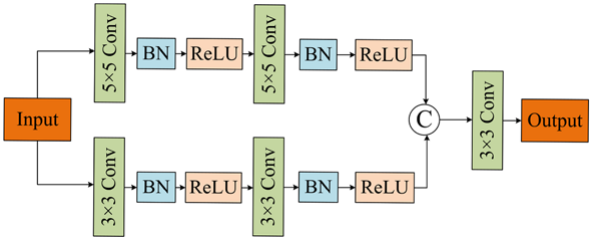
Multi-scale Residuals Block.

As shown in Fig. 5, the MSFR module employs four convolution units and two multi-scale residual blocks (MRB) for detecting multi-scale features and generating the final segmentation outcomes. Specifically, as shown in Fig. 6, a dual-branch network is constructed, where each branch uses a different convolutional kernel. To retain the original information of the input *X*, residual learning is used for obtaining the MRB output by adding *X* and fusing the multi-scale features.

## Experimental setup and results

This section gives details of the experimental environment and conditions, the experimental dataset, comparison against other methods, an ablation study, and experimental data analysis.

### Experimental environment and condition

The PyTorch framework was used to implement the proposed polyp segmentation model, using Res2Net^26^ as a backbone network. The number of channels in the convolutional layers outside the backbone network was uniformly set to 32 for the small model. To facilitate training and testing, each image was uniformly resized to 352 × 352. Resizing also allows a close approximation to realistic situations of colonoscopy, because the lens may rotate and zoom during colonoscopy examinations.

In addition, random expansion and erosion were also applied to the ground-truth labels to enhance generalizability. Model training was carried out using an initial learning rate of 10^–4^ and a polynomial learning rate decay^26^ with a factor of ($1-{(\frac{iter}{{iter}_{max}})}^{0.9}$). Two Tesla T4 16G GPUs were employed for model training.

### Datasets

In our experiments, a total of 2248 colonoscopy images were used where the images came from several datasets: Kvasir, CVC-ClinicDB, CVC-300, CVC-ColonDB, and ETIS. Moreover, 1450 images were randomly selected from the Kvasir and CVC-ClinicDB datasets to form the training dataset. The training data count represented about 55% of Kvasir and CVC-ClinicDB and 43% of all five datasets. The test dataset consisted of two parts: the non-training images of Kvasir and CVC-ClinicDB (denoted as T_1_) and all images of the three other datasets (CVC-300, CVC-ColonDB, and ETIS). And we will make a detailed comparison with other SOTA models on these datasets.

- The Kvasir^30^ dataset consists of 1000 polyp images, with image sizes varying from 332 × 487 to 1920 × 1072. The polyps in the images show blurred borders and low contrast with size and shape variations. This dataset was split into 900 images for training and 100 images for testing.
- The CVC-ClinicDB^31^ dataset has 612 images of sub-25 colonoscopy videos, from which 29 sequences were selected. The size of each image is 384 × 288. Training and testing were performed with 550 and 62 images, respectively.
- The CVC-300 dataset was selected from the EndoScene test dataset^32^, which contains 912 images from 44 colonoscopy sequences. Following Fan et al.^2^, CVC-300 was used as a test dataset with 60 test samples.
- The CVC-ColonDB^33^ dataset was mainly collected from 15 different colonoscopy sequences, with a total of 380 samples.
- The ETIS^34^ dataset contains 196 image samples collected from 34 colonoscopy videos. The size of each image is 1225 × 966, and this is the largest size among the explored datasets. This dataset is challenging since the polyp samples vary in size and shape, and some polyps are small and difficult to find.

### Comparative analysis with the state of the art

After the training dataset was completed for IECFNet, it was first evaluated on the T_1_ test dataset, and Table 1 shows the evaluation results. The results show that the IECFNet model significantly outperforms the other models.

**Table 1:** Comparison of experimental results with previous SOTA models on T1 dataset.

Dataset	Model	Mean dice	Mean IoU	MAE
KVASIR	U-Net	0.818	0.746	0.055
	U-Net++	0.821	0.743	0.048
	ResUNet++	0.813	0.793	–
	SFA	0.723	0.611	0.075
	PraNet	0.898	0.840	0.030
	Ours	0.907	0.856	0.028
CVC-CLINICDB	U-Net	0.823	0.755	0.019
	U-Net++	0.794	0.729	0.022
	ResUNet++	0.796	0.796	–
	SFA	0.700	0.607	0.042
	PraNet	0.899	0.849	0.009
	Ours	0.924	0.873	0.007

The predictive performance and the generalization ability between IECFNet and mainstream methods are compared. These methods are U-Net^22^, U-Net++^21,35^, ResUNet++, SFA, PraNet. U-Net and U-Net++ are the classical methods. SFA and PraNet are the state-of-the-art methods.

As Table 1 Comparison of experimental results with previous SOTA models on T1 dataset demonstrates, we provide a comprehensive comparison of our ensembles with the SOTA results reported in the literature IECFNet outperforms other models on CVC-ClinicDB for all metrics. Specifically, the IECFNet model has a mean Dice coefficient exceeding those of U-Net++ and ResUNet++ by 3% and 2.5%, respectively. This performance improvement is because that the proposed RFCE-e and RFCE-d modules can effectively extract rich fine-grained feature maps with high-level semantic information and low-level details. Moreover, the IECA module exploits fuzzy.

**Figure 7:**
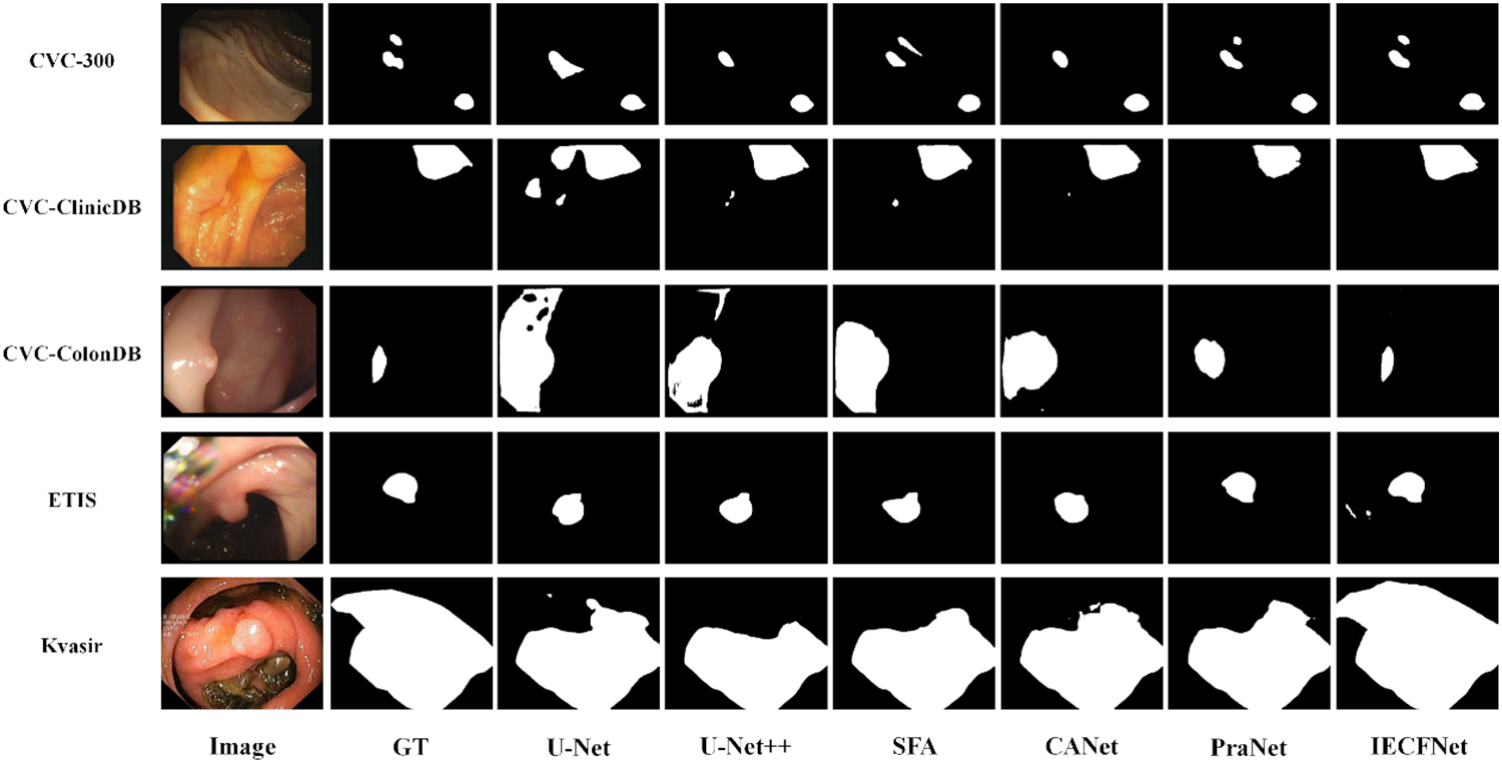
Comparison of qualitative results with the state-of-the-art methods on five different data sets.

Moreover, as mentioned earlier, the image sizes for the Kvasir dataset vary from 332 × 487 to 1920 × 1072, and these images show wide variations in polyp size and shape (see Fig. 7). The IECFNet model can deal with such large variations, and clearly outperforms the PraNet and SFA models on this dataset, thanks to the proposed multi-scale feature reasoning module with a two-branch structure for capturing multiscale features.

To evaluate the generalization capabilities of our model, comparative experiments were conducted on three datasets: CVC-300, CVC-ColonDB, and ETIS. The results are shown in Table 2. The ETIS dataset turned to be the most challenging dataset, and the IECFNet model achieved a mean Dice coefficient of 70.7% on this dataset, with a margin of improvement of 7.9% compared to the PraNet model. Figure 7 Comparison of qualitative results with the state-of-the-art methods on five different data sets shows sample qualitative results for the proposed model and the other state-of-the-art models on the five datasets.

**Table 2:** Comparison of experimental results with other SOTA models on ETIS, CVC-ColonDB and CVC-300 datasets.

Dataset	Model	Mean dice	Mean IoU	MAE
ETIS	U-Net	0.398	0.335	0.036
	U-Net++	0.401	0.344	0.034
	SFA	0.297	0.217	0.109
	PraNet	0.628	0.567	0.031
	Ours	0.707	0.632	0.016
CVC-COLONDB	U-Net	0.512	0.444	0.061
	U-Net++	0.483	0.410	0.064
	SFA	0.469	0.347	0.094
	PraNet	0.709	0.640	0.045
	Ours	0.775	0.632	0.019
CVC-300	U-Net	0.710	0.627	0.022
	U-Net++	0.707	0.624	0.018
	SFA	0.467	0.329	0.065
	PraNet	0.871	0.797	0.010
	Ours	0.912	0.850	0.005

As shown in Table 2, the IECFNet model achieved good performance on all metrics. For example, the mean Dice coefficient reaches 91.2% on the CVC-300 dataset, and IECFNet leads PraNet by 7.9% on the ETIS dataset. In contrast, the SFA model performance decreases sharply. Note that the images in the ETIS dataset have dimensions of 1225 × 966, the largest among the five datasets. Thus, one image in this dataset can have multiple challenging-to-segment polyps of different shapes and sizes (as shown in Fig. 7). Still, the evaluation results show that the IECFNet model has significant comparative advantages in dealing with multiple targets of different scales.

As shown in Fig. 7, the polyps in the third row are small, with blurred borders and low contrast, and thus these polyps are difficult to detect even with the naked eye. The IECFNet model still shows good segmentation results, while the segmentation results of the other methods are obviously not satisfactory. Also, the segmentation results of the 2nd, 4th, and 5th rows show that the IECFNet model slightly outperformed other models in dealing with some polyps with large differences in shape and appearance. In conclusion, for polyps with different shapes and sizes as well as for the multi-polyp cases, our IECFNet model demonstrated remarkably better results.

### Ablation experiments

To evaluate the effectiveness of the modules in the IECFNet model, three ablation experiments were conducted. The ablation studies were performed on the CVC-ClinicDB and ETIS datasets because CVC-ClinicDB was sampled for training purposes, but ETIS was not. The ablation experimental network model is still trained using the T1 training set in section IV.B of the chapter.

#### Ablation experiments on RFCA-e and RFCA-d

To verify the effectiveness of the RFCA-e and RFCA-d modules, a baseline IECFNet variant was constructed without the RFCA-e and RFCA-d modules (this model variant is denoted by "A").

Segmentation performance was evaluated using three metrics: mean Dice coefficient (mDice), mean intersection over union (mIoU), and mean absolute error (MAE). The experimental results are shown in Table 3. Moreover, the segmentation results can be qualitatively analyzed and visualized with a heatmap of the predicted segmentation probabilities for different image regions. Such a heatmap clearly shows the degree of model attention and confidence for different regions.

**Table 3:** Experimental results of RFCA module.

Dataset	Model	Mean dice	Mean IoU	MAE
CVC-ClinicDB	A	0.913	0.862	0.009
	IECFNet	0.924	0.873	0.007
ETIS	A	0.664	0.585	0.021
	IECFNet	0.707	0.632	0.016

#### Ablation experiments on IECA

Further ablation experiments were designed to demonstrate the effectiveness of the IECA module. Specifically, an IECFNet variant was built where the IECA module was replaced with a contextual attention (CA) module (this model variant is denoted by "B"). The output feature maps of the attention module were visualized for both settings to qualitatively verify the validity of the uncertainty regions. The segmentation results show that the IECFNet network with the IECA module easily identifies the uncertainty region that are closely related to the polyp boundaries. The experimental results are shown in Table 4.

**Table 4:** Results of ablation experiments of IECA modules.

Dataset	Model	Mean dice	Mean IoU	MAE
CVC-ClinicDB	B	0.920	0.871	0.008
	IECFNet	0.924	0.873	0.007
ETIS	B	0.714	0.625	0.015
	IECFNet	0.707	0.632	0.016

**Figure 8:**
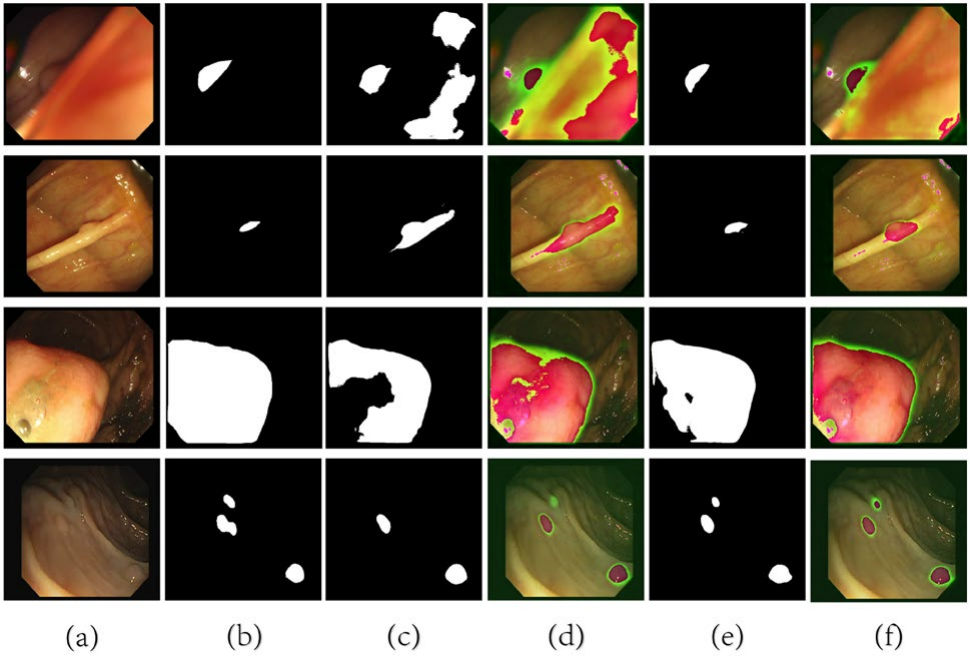
Ablation experimental model segmentation results. (**a**) Represents an image, (**b**) represents the ground-truth segmentation, (**c**) represents the segmentation result of the ablation experiment, (**d**) represents the segmentation heatmap of (**c**), (**e**) represents the IECFNet segmentation result, and (**f**) represents the segmentation heatmap of (**e**).

Also, the model with the IECA module performed better than the one with the CA module in terms of dealing with inaccurate localization and boundary blurring (as shown in the second row of Fig. 8). By comparing the heatmaps and feature maps of the segmentation results of the different models, the IECFNet model demonstrated better performance on small polyps with blurred boundaries, while the model with the CA module misidentified the normal tissues in the neighborhood of small polyps.

#### Ablation experiments on MSFR

In these experiments, the MSFR module was removed from the proposed IECFNet model, while the other model components remained the same (this model variant is denoted by "C"). The experimental results are shown in Table 5. The MSFR module actually made good use of the multi-scale information of the low-level features, and hence boosted the polyp segmentation performance.

**Table 5:** Results of MSFR module ablation experiments.

Dataset	Model	Mean dice	Mean IoU	MAE
CVC-ClinicDB	C	0.902	0.854	0.010
	IECFNet	0.924	0.873	0.007
ETIS	C	0.659	0.584	0.025
	IECFNet	0.707	0.632	0.016

As shown in the fourth row of Fig. 8, the IECFNet model is better than the model without the MSFR module in terms of dealing with the polyp size and shape variations and in handling multiple polyps. Actually, the IECFNet model effectively segments the small polyps in the upper left corner and improves the segmentation performance on polyps of larger sizes. This shows that the MSFR module of the IECFNet model is essential for dealing with polyps of different sizes and shapes.

## Conclusion

A new polyp segmentation network, called IECFNet, is proposed. This network first enhances regions of uncertainty by targeting saliency maps that are highly correlated with polyp boundaries. Then, the network refines low-level features using saliency-based attention maps. Finally, the network detects fused image features of different scales and performs multi-scale feature reasoning for accurate polyp detection. In the absence of edge labels, we use implicit edge regions for boundary representation. We also propose the receptive-field coordinate attention encoder (RFCA-e) module and the receptive-field coordinate attention decoder (RFCA-d) module to focus on the spatial features of the perceptual field. As well, a multi-scale feature reasoning (MSFR) module is proposed to get enhanced features after cross-layer feature fusion. Through a series of quantitative and qualitative experiments, the IECFNet model performs well compared to previous state-of-the-art methods.

## Data Availability

The dataset used in this study can be found at https://github.com/Zhangweiwei-ctgu.
